# 69742 miR-338-5p as a Biomarker of Neuropathic Pain After Spinal Cord Injury

**DOI:** 10.1017/cts.2021.632

**Published:** 2021-03-31

**Authors:** Jesse Kowalski, Nguyen Nguyen, Ricardo Battaglino, Scott Falci, Susan Charlie, Leslie Morse

**Affiliations:** 1 University of Minnesota; 2 Swedish Hospital; 3 Craig Hospital

## Abstract

ABSTRACT IMPACT: Identification of microRNA (miRNA) associated with neuropathic pain after spinal cord injury (SCI) will elucidate underlying epigenetic mechanisms contributing to its development and identify targets for intervention to optimize treatment strategies and outcomes. OBJECTIVES/GOALS: Approximately 70% of individuals with SCI experience neuropathic pain, which is refractory to pharmacologic intervention, and can reduce overall health and wellbeing. This study aims to identify predictive miRNA biomarkers of neuropathic pain after SCI to identify targets for the development of efficacious interventions. METHODS/STUDY POPULATION: Blood samples and clinical outcome measures were collected from adult participants with SCI with neuropathic pain (n = 28) and without neuropathic pain (n = 15). The sample population consisted of a mean age of 39 (SD = 12.12), 8 female (20%), with 13 classified as acute SCI (within 3 months post injury) and 30 as chronic SCI (> 3 years post injury). Pain presence, type, and intensity were assessed with the International Spinal Cord Injury Basic Pain Dataset (ISCIBPDS). Serum miRNA sequencing counts were produced from blood samples. Fold change and independent t-tests assessed differential expression between those with and without neuropathic pain, and those with chronic or acute SCI. Linear regression was performed to explore the relationship between miRNA expression and ISCIBPDS pain intensity ratings. RESULTS/ANTICIPATED RESULTS: In individuals with SCI, significant downregulation of expression of miR-338-5p was present in those with neuropathic pain compared to those without neuropathic pain (fold change = 0.81, p = 0.04). A significant relationship between expression of miR-338-5p and highest reported neuropathic pain intensity on the ISCIBPDS was identified (R2 = 0.15, F = 7.32, p < 0.01). Covariates of sex, age, and years post injury were not found to significantly influence the relationship between miRNA expression and ISCIBPDS intensity ratings. No significant differences in miR-338-5p expression were identified between participants with acute and chronic SCI, or with nociceptive pain ratings, demonstrating specificity of the relationship between miR-338-5p differential expression with pain of a neuropathic nature. DISCUSSION/SIGNIFICANCE OF FINDINGS: These findings, along with validated targets of miR-338-5p in the NF-KB neuroinflammatory signaling pathway, suggest that miR-338-5p may serve a neuroprotective role in modulating neuroinflammation, and that its downregulation may result in maladaptive neuroplastic mechanisms contributing to the development of neuropathic pain after SCI.

